# “I never made it to the pros…” Return to sport and becoming an elite athlete after pediatric and adolescent anterior cruciate ligament injury—Current evidence and future directions

**DOI:** 10.1007/s00167-017-4811-4

**Published:** 2017-11-29

**Authors:** Eric Hamrin Senorski, Romain Seil, Eleonor Svantesson, Julian A. Feller, Kate E. Webster, Lars Engebretsen, Kurt Spindler, Rainer Siebold, Jón Karlsson, Kristian Samuelsson

**Affiliations:** 1 Department of Health and Rehabilitation, Institute of Neuroscience and Physiology, the Sahlgrenska Academy, University of Gothenburg, Gothenburg, Sweden; 2 Sports Medicine Research Laboratory, Luxembourg Institute of Health, Strassen, Luxembourg; 3 Department of Orthopaedics, Institute of Clinical Sciences, the Sahlgrenska Academy, University of Gothenburg, Gothenburg, Sweden; 4 OrthoSport Victoria, Epworth HealthCare, Melbourne, VIC Australia; 5 School of Allied Health, La Trobe University, Melbourne, VIC Australia; 6 Oslo University Hospital and University of Oslo, Oslo, Norway; 7 OSTRC, The Norwegian School of Sports Sciences, Oslo, Norway; 8 Cleveland Clinic Sports Health Center, Garfield Heights, OH USA; 9 Institute for Anatomy and Cell Biology, Ruprecht-Karls-University, Heidelberg, Germany; 10 HKF, International Center for Hip, Knee, Foot Surgery and Sportstraumatology, ATOS Klinik, Heidelberg, Germany; 11 Department of Orthopaedics, Sahlgrenska University Hospital, Mölndal, Gothenburg, Sweden

**Keywords:** Anterior cruciate ligament, ACL, Pediatric, Adolescent, Return to sport, Sports, Reconstruction, Rehabilitation, PAMI

## Abstract

The management of anterior cruciate ligament (ACL) injuries in the skeletally immature and adolescent patient remains an area of controversy in sports medicine. This study, therefore, summarizes and discusses the current evidence related to treating pediatric and adolescent patients who sustain an ACL injury. The current literature identifies a trend towards ACL reconstruction as the preferred treatment option for ACL injuries in the young, largely justified by the risk of further structural damage to the knee joint. Worryingly, a second ACL injury is all too common in the younger population, where almost one in every three to four young patients who sustain an ACL injury and return to high-risk pivoting sport will go on to sustain another ACL injury. The clinical experience of these patients emphasizes the rarity of an athlete who makes it to elite level after a pediatric or adolescent ACL injury, with or without reconstruction. If these patients are unable to make it to an elite level of sport, treatment should possibly be modified to take account of the risks associated with returning to pivoting and strenuous sport. The surveillance of young athletes may be beneficial when it comes to reducing injuries. Further research is crucial to better understand specific risk factors in the young and to establish independent structures to allow for unbiased decision-making for a safe return to sport after ACL injury.

*Level of evidence* V.

## Introduction

We all have that one friend who keeps telling everyone around us the story of how he never made it to the top and competed with the best in the world. What happened? A simple side-cut that went wrong during sports participation at 12 years of age. A twist of the knee and within milliseconds the devastating outcome of an anterior cruciate ligament (ACL) tear. At first, you may think how common it is to read about athletes returning to sport after this injury. Without accusing your friend of lying, you conclude that he probably gave up or re-considered life choices, graduated and started a great career in economics or sports injury research. Sadly, however, the story of not reaching the top in sports after a pediatric or adolescent ACL injury may be based more on fact than fiction. The clinical experience of these patients emphasizes the rarity of an athlete who makes it to elite level after a pediatric or adolescent ACL injury, with or without reconstruction. To our knowledge, there are no studies of this topic. Nevertheless, this raises the question of whether current treatment regimens and patient education need to be revised?

## History and current evidence

An ACL tear is a common musculoskeletal injury, often sustained by athletes who participate in cutting and pivoting sports. A reconstruction of the ACL is often cited as the clinical standard to provide mechanical stability to the knee joint and a timely return to sport [1]. The available literature has shown that there is value to ACL reconstruction in patients after the age of 20 years [2–4]. However, there is limited literature about patients younger than 20 years of age.

Knee injuries have become more prevalent in children and adolescents over the last decade [5]. Studies based on reports from pediatric medical centers suggest that ACL injuries account for 6.3% of all sports injuries in children aged 5–12 and 10.6% among adolescents aged 13–17 [6]. Historically, it has been reported that the rates of ACL reconstruction increase more than eight times in adolescents aged 15–18, compared with younger patients in the 11- to 14-year category [7]. The rates of ACL reconstructions in children and adolescents are now increasing at a significantly higher tempo than in the adult population [7, 8]. This is particularly reflected in the Australian population, where the number of ACL reconstructions in patients under 15 years of age has increased by a factor of four over the past 15 years [9]. This is a matter of concern, since the management of ACL injuries in the skeletally immature patient remains an area of controversy in sports medicine [10]. It should also be remembered that an ACL injury at a young age should be regarded as a lasting injury, regardless of the type of treatment [11].

**Fig. 1:**
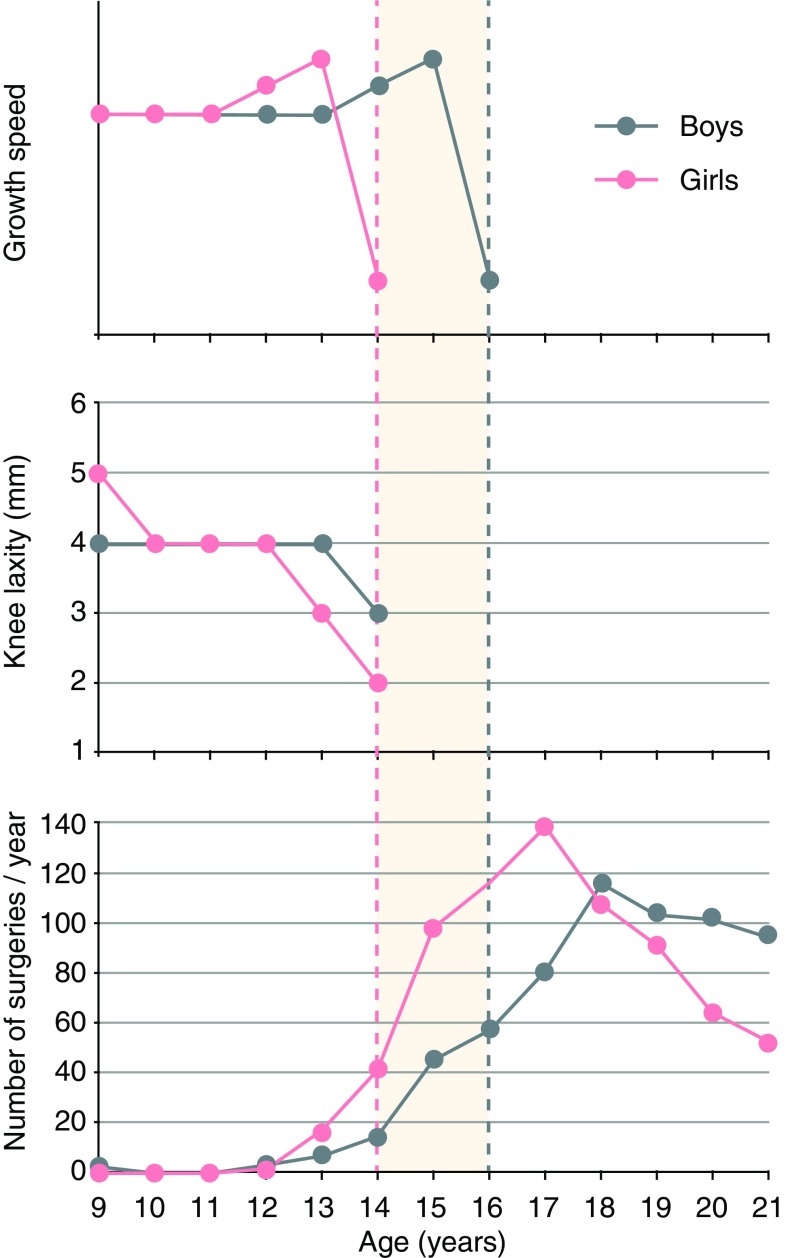
Presentation of the relationship between age, growth speed and cessation, knee joint laxity, and average number of ACL surgeries performed

The controversy can be partly explained by the fact that not all children and adolescents follow the average rates of skeletal growth. In general, the cessation of skeletal growth at the knee occurs at 14 years of age in girls and at 16 in boys. It is reported that, during the last few years of growth, the proximal tibial physis grows approximately 6 mm a year and the distal femoral physis contributes roughly 10 mm [12]. At the same time, knee laxity decreases, making the younger person’s knee stiffer (Fig. 1) [13, 14]. In addition, there is large interindividual variability in the timing of the cessation of growth and this is due to discrepancies between chronological and skeletal age. This means that each patient should be analyzed carefully and individually [10]. In the presence of an open physis, treatment options for ACL injuries become contentious, due to the risk of surgical physeal insults, which may result in limb-length discrepancies or limb malalignment [15, 16]. The anatomic placement of the graft during reconstructive procedures is also difficult due to the proximity of the growth plate to the aperture of the femoral tunnel [17].

**Fig. 2:**
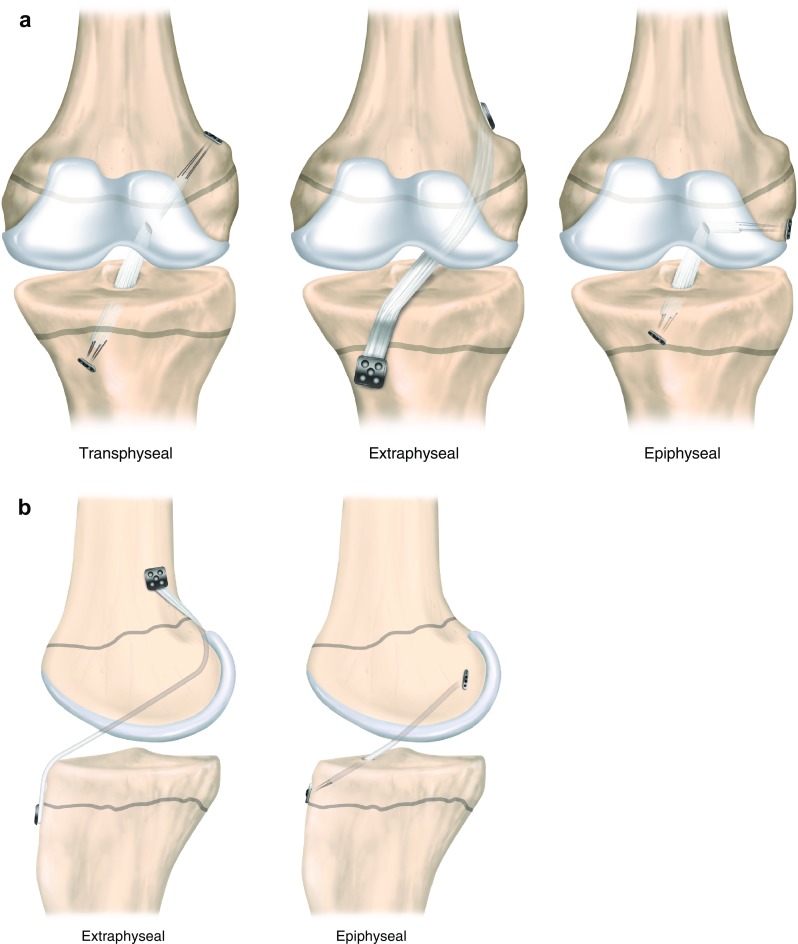
Representation of different pediatric ACL reconstruction techniques in an anterior view (**a**) and lateral knee view (**b**). Surgeons differentiate between transphyseal and physeal-sparing techniques. The ACL grafts are either placed within the epiphysis or turned around the physis. Many surgeons use different techniques on the femoral and the tibial side

Current options for treatment include non-surgical management with activity reduction or modification until physeal maturity, non-anatomic extra-articular procedures in which the graft is placed around the growth plate, physeal-sparing epiphyseal reconstruction where tunnels are placed within the tibial and femoral epiphysis, partial transphyseal procedures and transphyseal ACL reconstruction where tunnels are drilled through the growth plates (Fig. 2) [15, 18, 19]. Each treatment option has potential complications. The growth-related complication rate has been evaluated at < 2% [15], but this may be underestimated [20]. An evidence-based approach would facilitate management decisions with respect to the delicate balance between the need to stabilize the patient’s knee joint with reconstructive surgery and the requirement of avoiding complications due to physeal injuries [10]. ACL reconstruction in a skeletally immature patient is advocated to provide ligamentous knee-joint stability and potentially to protect the patient from subsequent concomitant injury. As with their adult counterparts, the absence of ligamentous knee-joint stability in children predisposes them to the risk of further meniscal and chondral injuries, thereby increasing the risk of early degenerative changes [21–23]. Additionally, the patient’s desire to return to strenuous or pivoting sport is frequently described as an indication for surgical treatment, similar to that in patients over 20 years of age [24, 25].

The current literature identifies a trend towards ACL reconstruction as the preferred treatment option for ACL injuries in the young, largely justified by the risk of further structural damage to the knee joint but also by the challenges of ensuring the compliance of the young patient to modify his/her activity level and the sub-optimal outcomes associated with non-surgical treatment [26–28]. The overall literature supports a non-surgical approach initially in patients < 13 years of age. As part of the non-surgical treatment, patients should be advised to modify their level of sports participation, refraining from pivoting sports [10, 19, 20]. Regular clinical assessments may be supplemented by an MRI of the knee to rule out secondary meniscus or cartilage lesions [20, 29, 30]. The first prospective cohort study with a minimum of 2 years of follow-up in 52 pre-puberty children (< 12 years) reported the appearance of secondary meniscal tears in 17% of ACL-injured patients [31]. This rate has to be contrasted to the high risk of second ACL injuries and other postoperative complications when it comes to surgical decision-making. Nonetheless, numerous surgical techniques and expert opinions on this topic have emerged [10]. This has been highlighted in a study by Moksnes et al. [20] who reported that there are substantial differences in preferred treatment algorithms and long-term follow-up procedures among orthopedic surgeons regarding the treatment of pediatric ACL injuries.

Typically, the surgical procedures involve a double autologous semitendinosus and gracilis graft of approximately 6–8 mm [15, 32]. Synthetic grafts and bone-patellar tendon-bone autografts should generally be avoided in the pediatric population because of the risk of growth disturbances [18, 33]. It is also recommended that drilling bone tunnels through a growth plate should be performed at a steep angle and a width of < 9 mm, to minimize the cross-sectional area of the tunnels with the aim of reducing the risk of disturbing the epiphyseal plate [10, 16, 19, 20, 34]. Additionally, the surgeon is able to confirm that tunnels are free of bone debris. It is also recommended that the pediatric patient who undergoes ACL reconstruction has annual standing long-leg radiographs to evaluate lower limb alignment and leg length discrepancy until skeletal maturity is reached at the level of the knee [29, 35].

The proportion of pediatric and adolescent patients who return to high-risk sports has been reported to be between 69 and 92% [9, 36–38]. However, a lower proportion of these patients appear to return to their pre-injury sport. At the same time, if their age is considered, these patients may well be involved in several sports, making it difficult to determine what actually constitutes the pre-injury sport [38]. In addition to this, the level of sports practice is constantly evolving in this young population. In most pivoting sports, children and adolescents do not reach their highest level of sports participation until late adolescence or early adulthood. As a consequence, the concept of return to sports is more complex in this young population as compared to their adult counterparts. In a recent publication by Webster et al. [39], two-thirds of adolescent patients who sustained an ACL injury and were able to return to their sport reported that they were able to perform as well as before the injury. At a follow-up after an average of 5 years, 48% of female patients were still participating in pivoting sports, as were 54% of males. The same study also reported fear of a new injury or study/work commitments as the primary reasons for never returning to or dropping out of sport.

Nevertheless, there are no studies explicitly investigating present or future level of participation in top level or elite sport for pediatric and adolescent patients. Can such a patient return to sport safely and fulfill his/her dream of becoming an elite athlete in a pivoting sport? If not, treatment should possibly be modified to take account of the risks associated with returning to pivoting and strenuous sport.

**Fig. 3:**
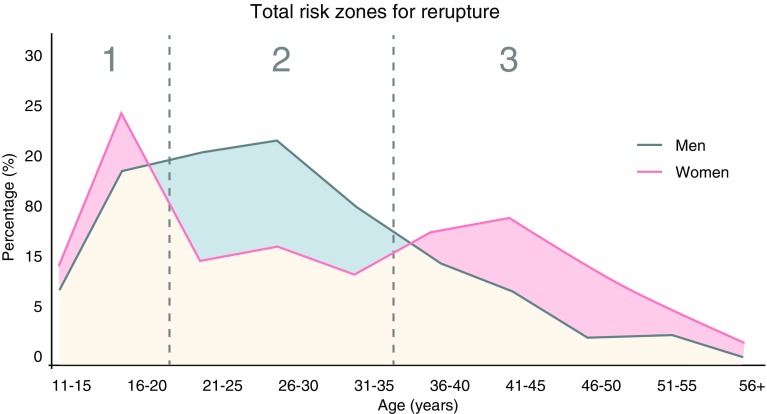
Distribution of anterior cruciate ligament re-ruptures across age and patient sex

Probably the most devastating complication following a return to sport is a second ACL injury, either a graft rupture or a contralateral ACL tear. Worryingly, a second ACL injury is all too common in the younger population (Fig. 3). In patients under 20 years of age, the probability of a second injury increases three to six fold [38]. Injury rates in younger cohorts have been reported to be as high as 30% in the literature [9, 38, 40, 41]. Aggregated results from reviews suggest that the younger patients who return to sports have a higher re-injury rate than those who do not [25, 42]. The current evidence strongly indicates that the risk of a second ACL injury is greatest within the first 2 years after returning to pivoting sports [9, 38, 43]. Overall, almost one in every three to four young patients who sustain an ACL injury and return to high-risk pivoting sport will go on to sustain another ACL injury [38].

Compared with an uninjured counterpart, it has been suggested that a young patient who returns to sport after ACL reconstruction runs a 30–40 times greater risk of ACL injury [42]. A report from the Norwegian National Knee Ligament Register confirms that age is a significant risk factor for both revision and contralateral ACL reconstruction with hazard ratios of 4.0 and 4.9, respectively, for the 15- to 19-year age group, compared with patients over 30 years of age [44]. Additionally, publications from the Swedish ACL Registry have reported that adolescent patients 13–19 years of age have the highest rates of early revision and that this group runs an almost three times greater risk of contralateral ACL reconstruction [2, 45]. These data suggest that the modification of sports participation, improved rehabilitation, the use of integrative neuromuscular training and validated criteria for a return to sport are important in the management of these patients to help them safely reintegrate into sport and reduce the risk of a second injury [46–49].

Interestingly, the literature suggests that the young active population appears to recover more quickly and transition back to sports earlier after ACL reconstruction compared with their older counterparts [6, 42, 50]. Nevertheless, a number of the younger patients have been reported to have remaining functional deficits and altered motor patterns [51, 52] in the reconstructed knee at the time of return to sport [53]. The current treatment approach of early accelerated rehabilitation and the expected timeframe of recovery of 9–12 months is potentially deleterious to younger athletes, as they may not be fully recovered [54]. Several authors have, therefore, suggested that waiting at least 2 years to reintegrate into high-risk sports will significantly benefit patients after ACL injury [9, 25, 55]. It must, however, be stressed that a prolonged waiting time for a return to sport may potentially further jeopardize these patients’ ability and willingness to return to the same level of sport after missing at least two seasons. In other words, this may have serious consequences for the young athlete’s immediate and future career.

Is it time to re-think the current treatment options for pediatric and adolescent patients with an ACL injury? Are we giving young patients, their parents and their coaches unrealistic expectations of returning to sport and the opportunity to become elite athletes? The literature tells us that most young patients are able to return to pivoting sport, but this is accompanied by the risk of a second ACL injury. Whether it is actually possible to become a world-class athlete after an ACL injury at a young age is a question that remains to be answered.

## Future directions

In the past decade, there have been changes in terms of the surgical techniques used for ACL reconstruction and postoperative rehabilitation, as well as significant advances in the identification of risk factors for graft rupture and contralateral ACL injury in younger patients [56]. It is important that these advances in management are translated into clinical practice. However, the recent literature and clinical experience have not shown any reduction in secondary ACL injury risk or improved outcome after treatment [42].

The recovery of baseline knee health and function should be the fundamental prerequisite, if possible, prior to a return to sport following ACL injury, independent of patient age. Modifications to return to sport guidelines have the potential to reduce the re-injury risk and hopefully improve future sports performance in the young athlete after ACL injury. Although various return-to-sport guidelines exist, there is still no consensus about, or validation of, these guidelines. This means that return  to  sport decisions are often based on a combination of time since surgery and personal experience [25, 50, 55, 56].

While studies that focus on pediatric and adolescent populations who return to their previous sport and report on the level of participation are rare, registry data involving large numbers of patients are beginning to emerge [2, 43, 57]. It is worrying that many young patients have high expectations in terms of future sports performance, but it is likely that only the occasional patient will have a future in elite sport. There is an urgent future need for more detailed data on return to sport for the high-risk younger population.

It is possible that there is some natural selection in play that puts some individuals at a higher risk of initial rupture and subsequent re-rupture. Intrinsic risk factors, including morphological variants such as a higher degree of laxity among certain patients [14], exist. However, we are currently unable to identify with any certainty the individual athlete at risk. The surveillance of youth athletes has previously been used in an attempt to identify risk factors for ACL and overload injuries in young athletes. Several general factors related to training and competition load have been identified and are helping us to understand the injury panorama among the young (Table 1) [58–62]. For instance, Malisoux et al. [61] showed that an increase in weekly training intensity was associated with an increased risk of injury in younger athletes, similar to what is found in their older counterparts [63]. This suggests that monitoring young athletes may be beneficial when it comes to reducing, and potentially preventing, injuries. One good example of an initiative of this kind is the European Society of Sports Traumatology, Knee Surgery and Arthroscopy (ESSKA) that launched the ESSKA Pediatric Anterior Cruciate Ligament Monitoring Initiative (PAMI) [20]. The goal of this initiative is to serve as a multinational network of centers dealing with this clinical problem to share knowledge, increase awareness and improve the understanding of injury occurrence, treatment approaches, the long-term effects, anatomy, biomechanics and reconstruction in the pediatric population. The ultimate aim of the initiative is to create an international pediatric ACL registry.

**Table 1:** Risk indicators for anterior cruciate ligament injuries and other overload injuries identified from surveilling youth athletes

> 10 days without rest
> 50% intense training sessions
Competition despite injury
> 7 days indication of injury / symptom
Recurrent injuries

This type of further research is crucial to obtain a better understanding of specific risk factors in the young and to establish independent structures to allow for unbiased decision-making for a safe return to sport after ACL injury. There is also a lack of information on these patients’ future quality of life and premature osteoarthritis development that needs to be addressed.
